# Immunohistochemistry versus PCR Technology for Molecular Subtyping of Breast Cancer: Multicentered Expereinces from Addis Ababa, Ethiopia

**DOI:** 10.15430/JCP.2023.28.2.64

**Published:** 2023-06-30

**Authors:** Dessiet Oma, Maria Teklemariam, Daniel Seifu, Zelalem Desalegn, Endale Anberbir, Tamrat Abebe, Solomon Mequannent, Solomon Tebeje, Wajana Lako Labisso

**Affiliations:** 1 Department of Medical Biochemistry, College of Health Sciences, Addis Ababa University, Addis Ababa, Ethiopia; 2 Department of Biochemistry, University of Global Health Equity, Kigali, Rwanda; 3 Department of Microbiology, Immunology, and Parasitology, School of Medicine, College of Health Sciences, Addis Ababa University, Addis Ababa, Ethiopia; 4 Department of Surgery, School of Medicine, College of Health Sciences, Addis Ababa University, Addis Ababa, Ethiopia; 5 Department of Pharmacology, School of Pharmacy, College of Health Sciences, Addis Ababa University, Addis Ababa, Ethiopia; 6 Department of Pathology, College of Health Sciences, Addis Ababa University, Addis Ababa, Ethiopia

**Keywords:** Breast cancer, Molecular subtypes, Immunohistochemistry, Reverse transcription polymerase chain reaction

## Abstract

The application of immunohistochemistry (IHC) for molecular characterization of breast cancer (BC) is of paramount importance; however, it is not universally standardized, subject to observer variability and quantifying is a challenge. An alternative molecular technology, such as endpoint reverse transcription (RT)-PCR gene expression analysis, may improve observer variability and diagnostic accuracy. This study was intended to compare IHC with the RT-PCR based technique and assess the potential of RT-PCR for molecular subtyping of BC. In this comparative cross-sectional study, 54 BC tissues were collected from three public hospitals in Addis Ababa and shipped to Gynaecology department at Martin-Luther University (Germany) for laboratory analysis. Only 41 samples were qualified for IHC and RT-PCR investigation of estrogen receptor (ER), progesterone receptor (PR), human epidermal growth factor receptor 2 (HER2), and Ki-67 protein expression analysis. Kappa statistics was used to assess the concordance between the two techniques. The overall percent agreement between RT-PCR and IHC was 68.3% for ER (positive percent agreement [PPA] 71.1%; negative percent agreement [NPA] 33.3%), 39.0% for PR (PPA 14.3%; NPA 92.3%), and 82.9% for HER2 (PPA 62.5%; NPA 87.9%). Cohen’s κ-values of 0.018 (< 0.20), 0.045 (< 0.200), and 0.481 (0.41-0.60) were generated for ER, PR, and HER2, respectively. Concordance for molecular subtypes was only 56.1% (23/41) and 0.20 kappa value. IHC and endpoint RT-PCR techniques have shown to be discordant for 43% samples. Molecular subtyping using endpoint RT-PCR was fairly concordant with IHC. Thus, endpoint RT-PCR may give an objective result, and can be applied for BC subtyping.

## INTRODUCTION

Breast cancer (BC) is the most prevalent form of cancer in women and the main cause of cancer mortality worldwide. Recent reports indicate that female BC has overtaken lung cancer as the most commonly diagnosed cancer, with an estimated 2.3 million new cases (11.7%), followed by lung cancer (11.4%). The same report confirmed that 684,996 (6.9%) new deaths occurred globally in the same year [1-3]. BC is one of the most common public health challenges in Africa, with an estimated 186,598 new cases and 85,787 deaths annually [2]. The incidence of BC in Africa is lower than developed countries; however, significantly high mortality is observed. This could be due to many reasons including racial, biological, socio-economic and socio-cultural factors [3,4], in addition to limited awareness of the general population about BC and lack of advanced diagnostic and treatment means in the continent. In Sub-Saharan African countries including Ethiopia, BC is the most diagnosed cancer among women [3,5]. In Ethiopia, according to GLOBOCAN 2020 report, BC incidence accounts 20.9% of all cancers in female, followed by cervical cancer 9.6% [6].

BC is a heterogeneous disease with various molecular subtypes and biological features, resulting in a different treatment response and clinical outcomes. Depending on molecular signature, it is classified into five different molecular subtypes; namely, luminal-A, luminal-B, human epidermal growth factor receptor 2 (HER2)-positive, basal-like and normal breast-like breast cancers [7-9]. This is based on the expression pattern of the corresponding receptors on the surface of BC cells. More recent discovery by using gene expression profiling revealed an additional revealed an additional subtype of BC called Claudin-low BC. This has been characterized by absence of expression of the three breast cell receptors (estrogen receptor [ER], progesterone receptor [PR], and HER2). Breast cancers that lack the three cell surface receptors are also called triple negative breast cancers (TNBC). In addition to displaying the TNBC phenotype, Claudin-low BCs also show limited expression of Ki-67. Generally, only a small proportion of TNBCs are Claudin-low; that is, not all TNBCs are Claudin-low types. Claudin-low breast tumors were linked to a young age, a higher tumor grade, a larger tumor size, widespread lymphocytic infiltrate, and a restricted tumor margin, as well as poor survival [10]. Many literatures demonstrate that Claudin-low is not merely a subtype similar to the intrinsic subtypes (luminal-A, luminal B, normal breast-like, basal-like, and HER2-positive) but it is a complex extra phenotype that may amalgamate BCs of diverse intrinsic subtypes [11].

However, it is unaffordable to classify BC into molecular subtypes by using DNA microarray methods. As a result, immunohistochemistry (IHC) is widely recognized as a suitable replacement for gene expression profiling techniques [12]. Yet, routine BC diagnosis by IHC has a limitation for many laboratories, especially in low settings. Thus, the limitations of surrogate IHC for molecular subtyping of BC emphasize the need for better technique like end point reverse transcription PCR (RT-PCR). The latter has several advantages over IHC. It reduces the intra- and inter-observer based bias and does not require experienced pathologists for result interpretation. RT-PCR is feasible, can be standardized and is suitable to implement in low settings. The endpoint RT-PCR technique also enables the use of fresh frozen tissue samples which overcomes the challenges posed by fixation and paraffin embedding [13,14].

Regular BC molecular subtyping is not widely performed in Ethiopian hospitals. This is because IHC is unavailable and expensive technique, resulting in a gap in the quality of BC management. In this study, we compared IHC and endpoint RT-PCR for molecular subtyping of BC based on the ER, PR, HER2, and Ki-67 status using fresh frozen and formalin-fixed paraffin-embedded (FFPE) tissues samples from BC patients. Thus, this study was intended to validate the application of feasible, available, standardized, cost-effective and more accurate molecular techniques for subtyping of BC in routine diagnosis. The potential application of endpoint RT-PCR to improve the accuracy of the BC diagnosis was investigated.

## MATERIALS AND METHODS

### Study design and population

A comparative cross-sectional study was conducted in three hospitals in Addis Ababa: Tikur Anbessa Specialized Hospital, St. Paul’s Hospital Millennium Medical College, and Yekatit 12 Hospital Medical College from April, 2019 to February, 2021. The study population included breast cancer patients who underwent surgery in these hospitals during the study period. Breast cancer patients who have been treated with neoadjuvant therapy, with pregnancy, those with a history of any other malignancy, and age < 18 were excluded from the study.

### Socio-demographic data and tissue sample collection

Socio-demographic data were collected by trained nurses with structured questionnaire. Clinico-pathological characteristics of the study participants were collected from patients’ medical records. A total of 54 breast cancer tissue samples were collected from breast cancer patients that underwent surgery at the three hospitals. The collection of tumor tissue was done by attending surgeons in these hospitals. Portions of the solid tumors, free of fat, connective tissue, debris, and blood, were cut into pieces of approximately 3 × 3 × 3 mm and it was transported using icebox. Then, it was rapidly frozen at –80°C in deep freezer till further laboratory analysis.

### Tissue sectioning and haematoxylin and eosin staining

Breast cancer tissue was washed with PBS, fixed in Roti^®^ Histofix (Carl Roth) for 24 hours, dehydrated using ASP300 Tissue Processor (Leica) and embedded in paraffin. A series of 4 µm thick sections were cut for staining. Wax was removed from paraffin embedded tissue sections with the aid of 2× Roti^®^ Histol (Carl Roth) for 5 minutes. Then, the tissue was rehydrated with the decreasing alcohol series (2× 100%, 2× 96%, and 2× 80% EtOH), each for 3 minutes. Sections were then stained in haematoxylin for 5 sec, gently washed in tap water for 10 minutes and then stained in eosin for approximately 20 seconds. Tissue was dehydrated with an increasing alcohol series (2× 80%, 2× 96%, and 2× 100% EtOH). After immersing 2× for 5 minutes in Roti^®^ Histol (Carl Roth) slides were mounted in Pertex (Carl Roth) for microscopic observation.

### IHC

The breast cancer tissue was rehydrated after de-paraffinizing the embedded tissue sections. Microwave antigen retrieval was performed using unmasking solution (Vector Laboratories) for 9 minutes. After a cooling period of at least 15 minutes, slides were washed in distilled water. Endogenous peroxidase reactivity was blocked by incubating the slides in 3% H_2_O_2_ (Merck KgaA) for 20 minutes. After washing with water and PBS (Invitrogen GmbH) for once and twice, respectively, incubation with 5% serum in PBS for 1 hour was carried out to block unspecific antibody binding. Primary antibodies (R&D Systems) were diluted to a desired concentration in 3% serum in PBS and incubated. In all cases, the primary antibody was diluted to a factor of 1:500, goat serum was used for blocking and incubation was for 1 hour at room temperature. The slides were washed three times with PBS to remove non-specifically bound primary antibody. Biotinylated secondary antibody was diluted (1:200) and incubated for 1 hour at room temperature. After washing, VECTASTAIN^®^ Elite ABC solution (Vector Laboratories) was added and afterwards, slides were incubated with 3,3’-diaminobenzidine tetrahydrochloride (Vector Laboratories) until suitable brown staining is developed. Slides were finally counterstained with hematoxylin and mounted in Pertex for microscopic observation.

### IHC interpretation

The BC tissue is considered positive for ER and PR if at least 10% of cancer cells showed positive nuclear staining of any intensity according to the American Society of Clinical Oncology/College of American Pathologists (ASCO/CAP) guideline [15]. HER2 was graded based on recommendations from Allred scoring system as described in the ASCO/CAP as 0, 1+, 2+ or 3+. Scores of 0 and 1+ was considered negative and 3+ was considered positive. When a score of 2+ (weakly positive or equivocal) was found, additional Chromogenic in situ hybridization or FISH testing was done to establish HER2 gene amplification status [16]. Ki-67 grading was based on the proportion of positively stained cancer cell nuclei out of all cancer cell nuclei in the tissue section and the result was provided as a percentage ranging from 0 to 100%. To evaluate the expression of Ki-67, the positively stained nuclei of cancer cells was counted and expressed as the percentage designated the Ki-67 index. The Ki-67 index was considered low if the value is below 20% but high if it is equal or greater than 20% of cancer cells [17].

### Extraction of RNA from FFPE tissue

The RNA extraction was carried out by miRNeasy Mini Kit (QIAGEN GmbH) which combines phenol/guanidine-based lysis of samples and silica membrane–based purification of total RNA. A piece of tissue was excised from FFPE tissue samples. Multiple sectioning of an 8 µm size was produced using microtome and was used for RNA extraction. Tissue samples were homogenized in QIAzol Lysis Reagent (Qiagen GmbH) and after addition of chloroform, the homogenate was separated into aqueous and organic phases by centrifugation. RNA was partitioned to the upper, aqueous phase, while DNA was partitioned to the interphase and proteins were partitioned to the lower, organic phase or the interphase. The upper aqueous phase was extracted, and ethanol was added to provide appropriate binding conditions for all RNA molecules from 18 nucleotides upwards. The sample was then applied to the RNeasy Mini spin column (QIAGEN GmbH-Germany), where the total RNA binds to the membrane and phenol and other contaminants were efficiently washed away. High-quality RNA was then eluted in RNase-free water [18].

### Cell lines and gene expression analysis using endpoint RT-PCR

The BT474 and BT-20 breast cancer cell lines were used for optimization. For each run, a positive (BT474) and a negative (BT-20) control cell lines with known estrogen receptor 1 (ESR1), progesterone receptor (PGR), and human epidermal growth factor receptor 2 (ERBB2) expression were used [19]. RPL37-A was used as a reference gene. The sequences of primer pairs (forward and reverse) for the corresponding gene of interest are indicated in the Table 1.

Following the optimization on breast cancer cell lines, the end point RT-PCR experiment was done on the isolated RNA. The extracted RNA of each breast cancer cell lines and patient biopsy samples was used to synthesize cDNA using the Biozym cDNA synthesis kit (QIAGEN GmbH-Germany). To choose the optimum cDNA concentration amount required for PCR experiment, it was started with 100 ng, 50 ng, and 25 ng cDNA. Moreover, variable PCR conditions including the number of cycles for each gene of interest was checked to set the optimum conditions for the establishment of the method. The determined optimum PCR condition was set as follows: Denaturation: 95°C (2 minutes); Annealing: 62°C (20 seconds); Extension: 72°C (20 seconds) with a PCR cycles of 35. The PCR products were visualized through agarose gel electrophoresis bands using Image Quant LAS 4000 luminescent analyser (QIAGEN GmbH-Germany).

### Statistical analysis

All statistical analyses were performed using IBM SPSS version 25 (IBM Corp.). Overall percent agreement (OPA), positive percent agreement (PPA) or sensitivity and negative percent agreement (NPA) or specificity was calculated using 2 × 2 contingency tables. Kappa statistic with 95% two-sided confidence intervals was calculated to estimate the overall agreement between the endpoint RT-PCR and IHC, with κ-values categorized into poor (< 0.2), fair (0.2-0.4), moderate (0.4-0.6), good (0.6-0.8) and very good (> 0.8). Mean and median was used for age of the study participants. Statistical significance was defined as *P*-value less than 0.05. Only representative pictures of histopathology and immunohistochemistry were demonstrated.

### Ethical clearance

This study was conducted in accordance with the Declaration of Helsinki. It was reviewed and approved by the Department of Medical Biochemistry Ethics and Research Committee, Addis Ababa University, College of Health Sciences, School of Medicine with a protocol number: SOM/BCHM/129/2006. Written informed consent was obtained from all study participants. The study participants were also informed of the privacy and confidentiality of their personal information; that is, the study data will not be disclosed to a third party by any means.

## RESULTS

### Socio-demographic characteristics

Assessment of ER, PR, HER2, and Ki-67 using endpoint RT-PCR and IHC were performed on BC tissue samples from 41 of 54 study participants. The remaining 13 samples were excluded because FFPE tissue was unqualified for the study and fresh frozen tissue sample was cancer-free. There were 40 women among the 41 study participants, with a median age of 38.5 years (range 28-71) and a mean age of 42.48 years (standard deviation 9.89). Only one (2.4%) of the study participants was under the age of 30, while 20 (48.8%) were 30-39 years old. More than half (22, 53.7%) of the study participants live in urban area. Educational status of the study participants indicated that 14 (34.1%) had completed primary school, nine (22.0%) were illiterate, seven (17.1%) had completed college, and the remaining four (9.80%) had completed high school. The socio-demographic characteristics of the study participants are summarized in Table 2.

### Exposure status of study subjects for breast cancer risk factors

Premenopausal women made up 24 (58.5%) of the study participants. Five (12.2%) of the patients had a family history of breast cancer, and six (14.6%) had radiation exposure to the chest. Physical activity, cigarette smoking, and alcohol consumption were 4 (9.6%), 0 (0.0%), and 1 (2.4%), respectively (Table 3).

### Clinico-pathological characteristics of the study subjects

The frequency of clinico-pathological characteristics of the study participants is summarized in the Table 4.

### Evaluation of ER, PR, HER2, and Ki-67 using IHC

There were 38 (92.7%) ER-positive, 27 (65.9%) PR-positive, and 8 (19.5%) HER2-positive breast cancers. There was high Ki-67 expression (n = 25, 61.0%) observed in this study (Table 5).

### Evaluation of ER, PR, and HER2 using endpoint RT-PCR

**Figure 1:**
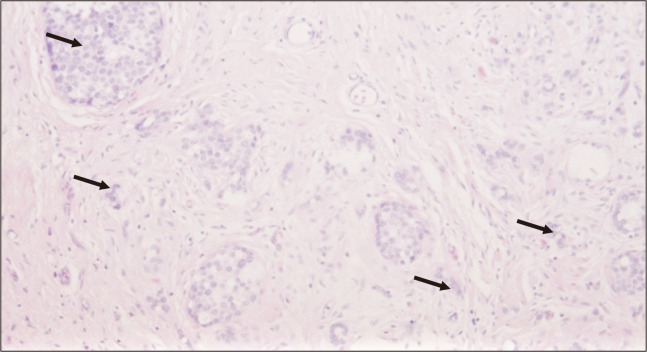
Representative image of breast cancer tissue stained with haematoxylin and eosin (20×). Arrows depict multinucleated cells with mitotic figures.

**Figure 2:**
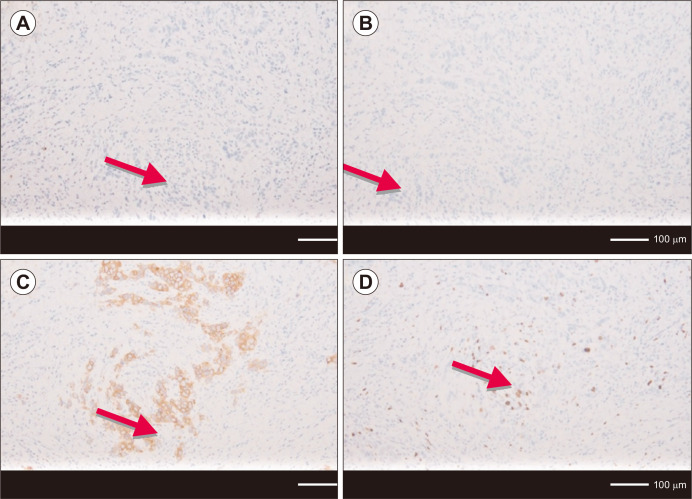
Representative images of IHC of ER, PR, HER2 and Ki-67 in human breast cancer tissue (20×). Arrows showing abundantly stained areas for different receptors in breast cancer tissues. Breast cancer tissue was stained with antibodies specific for ER, PR, HER2 and Ki-67. Images depict ER-positive (A), PR-positive (B), HER2-positive (C) and Ki-67-positive (D). IHC, immunohistochemistry; ER, estrogen receptor; PR, progesterone receptor; HER2, human epidermal growth factor receptor 2.

**Figure 3:**
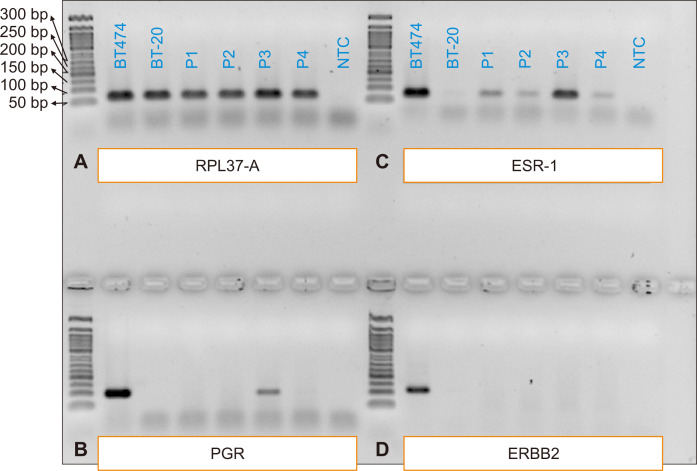
Gel document of endpoint RT-PCR. (A) RPL37-A (reference gene); BT474: positive control, BT-20: negative control, P1-P4 patient samples, and NTC. (B) ESR-1 gene; BT474: positive control, BT-20: negative control, P1-P4 positive sample. (C) PGR gene; BT474: positive control, BT-20: negative control, P3 positive sample, P1, P2 and P4 negative sample. (D) ERBB2 gene; BT474: positive control, BT-20: negative control, P1-P4 negative sample. RT-PCR, reverse transcription PCR; NTC, no template control.

There were 29 (70.7%) ER-positive breast cancers, five (12.2%) PR-positive breast cancers, and 9 (22.0%) HER2-positive breast cancers (Table 6). Figures 1-3 shows representative images of positive endpoint RT-PCR for ER, PR, and HER2.

### Molecular subtyping based on IHC and endpoint RT-PCR

Based on IHC using the expression pattern of ER, PR, and HER2, luminal-A (ER+ and/or PR+, HER2–) was more prevalent (75.6%) and HER2-positive (HER2+, ER–, and PR–) was less prevalent (2.4%). However, according to 2013 St. Galen international expert consensus, which includes Ki-67, the luminal-B subtype was the most prevalent (58.5%), followed by luminal-A (34.1%), TNBC (4.9%), and HER2-positive (2.4%).

On the other hand, using endpoint RT-PCR, the molecular subtyping was based on the expression of hormone receptors and HER2 because Ki-67 was not evaluated by endpoint RT-PCR. We found that luminal-A was most prevalent and HER2-positive was least prevalent (Table 7).

### Concordance between IHC and endpoint RT-PCR based on ER, PR, and HER2 status

Comparison of IHC and endpoint RT-PCR results of molecular subtyping of breast cancer was done based on ER, PR, and HER2. ER protein and ESR1 gene expression showed a slight overall agreement of 68.3% (PPA 71.1%; NPA 33.3%) while 13 cases were discordant, with 11 cases each positive by IHC and negative by endpoint RT-PCR; while 2 cases were negative by IHC and positive by endpoint RT-PCR. Overall agreement for PR protein and PGR gene expression was 39.0% (PPA 14.3%; NPA 92.3%). For PGR, 25 cases were discordant, with 24 cases each positive by IHC and negative by endpoint RT-PCR, while 1 case was negative by IHC and positive by endpoint RT-PCR. HER2 protein and ERBB2 gene expression showed moderate overall agreement with OPA 82.9%, PPA 62.5%, and NPA 87.9%. Table 8 shows the summarized concordance between IHC and endpoint RT-PCR based on ER, PR, and HER2 status.

### Concordance between IHC and endpoint RT-PCR based on molecular subtypes

IHC and endpoint RT-PCR molecular subtypes showed 56.1% overall concordance and κ of 0.20 (fair agreement). Out of 31 cancers that were classified as luminal-A by IHC, only 19 (61.3%) were similarly classified, the 12 discordant cases being classified as either luminal-B (n = 4, 12.9%) and TNBC (n = 8, 25.8%). Out of the 7 cases that were classified as luminal-B by IHC, three (42.9%) were similarly classified, the 4 discordant cases being classified as luminal-A (n = 2, 28.6%), HER2+ (n = 1, 14.3%) and TNBC (n = 1, 14.3%). The HER2+ molecular subtype was not similarly classified. Out of 2 samples which were classified as TNBC by IHC, one (50.0%) was similar and one was discordant case classified as luminal-A (50.0%). Table 9 shows the summarized concordance of molecular subtyping using IHC and endpoint RT-PCR.

## DISCUSSION

The management strategy and treatment outcome of BC are partly influenced by the appropriate, accurate and sensitive diagnostic means. The diagnostic modalities applied for BC detection vary from laboratories to laboratories based on the available resources in a given setting [20]. Thus, revisiting the existing diagnostic techniques of BC is essential to increase the accuracy and sensitivity in addition to minimizing the cost incurred on the patient and health system.

In addition to imaging modalities, there are several potent molecular diagnostic means that can provide patients and clinicians with personalized diagnostic information and allow targeted therapy. IHC is one of such modalities which plays a greatest role in determining BC. IHC with the advanced image analysis technologies has become instrumental to offer a direct visualization of tissue antigens using specific antibodies. However, it has been suffering from a number of limitations [20,21]. One of the major downsides of IHC is absence of objective and quantitative measurement of target antigens under examination. Thus, reinvestigating an alternative molecular diagnostic means for BC is highly needed. To this end, we evaluated the potential use of the endpoint RT-PCR technique in substituting the conventional IHC technique for BC detection, particularly in low settings [13,14,21].

The socio-demographic data indicated that the age of study participants ranged from 28 to 71 years, with the mean age of 42.4. About 58% of the study participants were pre-menopausal. This is in parallel with the previous reports in Ethiopia [22,23]. BC in Sub-Saharan Africa is reported to occur in younger age and show more aggressive features with histologic characteristics of high-grade and triple-negative molecular subtypes (ER, PR, and HER2 negative expression) [24-27]. In contrast, BC in developed countries is reported to occur at advanced ages of the patients [28]. The predominant appearance of BC in younger population in Ethiopia and other African patients might be due to environmental or genetic factors, such as rapid urbanization, and adoption of unhealthy lifestyles, as well as the fact that African countries have a higher number of younger populations [26,29]. Similar study on South African women showed the use of oral and injectable hormonal contraceptives that might be one of the causes of BC at young age [30]. However, an explicit study is needed to define the deriving cause of BC in younger population in African women.

As evudenced from the global reports, the invasive ductal carcinoma was the predominant histological type (90.2%) in this study [24,25,31]. Our study also shows that more than half of the BC cases were detected at advanced stages. Furthermore, stage III (51.2%) and grade III (56.1) are the predominant proportions of the cases in this study. Similar study in other parts of the country showed that most of BC cases were observed at advanced stages where patients cannot benefit from the available treatment means [22,32]. Consistent with our finding, researchers from other African countries demonstrated high prevalence of stage III and grade III BCs [24,26]. This is attributed to the fact that BC patients are presented at advanced stage of the disease due to lack of awareness, a low educational level, undermining symptoms, cultural beliefs, lack of medical facilities and trained personnel [27,32]. This, in turn, indicates that more effort is needed to curve the escalating incidence and mortality of BC in the unprivileged groups of people in Africa.

We found that luminal-B (58.5%) was the most prevalent molecular subtype of breast cancer in our study. Similar independent studies in Chinese women and European women revealed 54.3% and 57.1% luminal-B molecular subtypes of BC, respectively [33-36]. Recent studies from Mozambique (49%), South Africa (42.2%), and Kenya (35.8%) also showed relatively predominant luminal-B molecular subtype of BC [27,37,38]. Previous studies in Ethiopian women also demonstrated high prevalence of the hormonal positive breast cancers [23,32]. This is unlike the Western African and the African-American BC patients where the hormonal (ER and PR) and HER2-negative BCs are reported to be predominant [22,29,39]. Further investigation is needed to explain the clear causes of variation in landscapes of intrinsic molecular subtypes of BC between the Western and Eastern African populations, and the world in general.

However, based on expression patterns of ER, PR, and HER2, luminal-A is the most frequent subtype (75.6%) followed by luminal-B (17.1%), TNBC (4.9%) and HER2-positive (2.4%). Previous studies in Ethiopian BC patients also showed high prevalence of luminal-A (40%-54%) followed by luminal-B (22%-26%) subtypes [23,32]. Our result is also considerably in agreement with studies done in Algeria (50.6%) and Guinea (34.5%) where high prevalence of luminal-A and luminal-B molecular subtypes were reported [25,40]. The later discrepancy might be due to differences in the classification methods between studies and there could be interlaboratory variations in assessment of the expression of ER, PR, HER2, and Ki-67 [33].

The core objective of this study was to investigate the potential of endpoint RT-PCR in substituting the conventional IHC methods for determining intrinsic molecular subtypes of BC. Here, we report the existence of poor concordance between endpoint RT-PCR and IHC methods for determining ER and PR, with kappa value of less than 0.20 (OPA 68.3% and 39.0%, respectively). We observed moderate agreement between IHC and endpoint RT-PCR in detecting HER2 with kappa value of 0.481 and OPA 82.9%. This finding disagrees with previous studies which showed good to high concordance with highest OPAs for ER (91.8%-99%), PR (82.5%-94%), and HER2 (86%-97%) while PR and HER2 showed high specificity 92.3% and 87.9%, respectively [21,22,41,42]. The discrepancy could be due to variations in inter-laboratory methods, sample size differences, and processing techniques.

The molecular subtyping with IHC and endpoint RT-PCR showed fair agreement with OPA and kappa values of 56.1% and 0.20, respectively. This is relatively minimal when compared to other findings that showed 91.6% concordance with κ value of 0.885 [43]. Generally, 31 samples out of 41 were positive for luminal A by IHC technique, from which only 19 samples (61.3%) were positive for the same variable by the endpoint RT-PCR. This is in parallel to other study that showed fair concordance between IHC and endpoint RT-PCR for luminal-A subtypes of breast cancer classifications [41]. Furthermore, IHC and endpoint RT-PCR are concordant at 42.9% and 50% level for both luminal-B and TNBC, respectively. Similar studies by Janeva et al. [34] and Wirtz et al. [41] indicated the concordance rate of IHC and endpoint RT-PCR of 61.2%, and 85.7% for luminal-B and 87.1% and 62.5% for TNBC. In this study, it was not possible to compare the potential of endpoint RT-PCR for substituting IHC because only one sample was positive for the HER2 and HER2-positive subtype. Surprisingly, 79.6% and 100% concordance of IHC and RT-PCR technology was reported from elsewhere [41].

The main explanation for the relatively less OPA might be due to the application of different research approaches. Here, we used endpoint RT-PCR to analyze gene expression (which is qualitative), whereas other studies used RT-qPCR. Variability in the management of pre-analytical samples and the scoring technique could also be an important factor for the variation in results [21]. Another reason for discrepancies in ER, PR, and HER2 assessment using both methods, as well as misclassification of breast cancer molecular subtyping using both methods, is that IHC may be biased in favour of selected or representative tumor areas, whereas endpoint RT-PCR gene expression assays frequently analyse gene levels in the entire tumor mass (which is a reflection of the average gene expression in the entire tissue slice) [34,42,43].

Furthermore, discrepancies between mRNA expression and protein expression as measured by endpoint RT-PCR and IHC could be due to imbalance between post-transcriptional and post-transnational modifications. As mRNAs are less stable than proteins, this could be a contributing factor to low mRNA expression despite high protein expression [35,42].

The intention of this study was to compare IHC and PCR basetechnology for BC molecular subtyping. Accordingly, the endpoint RT-PCR and IHC results are slightly concordant. We suggest that endpoint RT-PCR might have a promising role in substituting IHC for breast cancer diagnosis in low-resource countries, such as Ethiopia. However, further investigation, with higher sample size and better sample processing and handling techniques, is needed to come to conclusive results.

Due to limited resources, we applied only small proportions of sample size and this could be one of the main reasons for the discrepancies in the results. Samples were transported to other laboratories in Germany. Thus, there could be damaging of samples during preparation, processing and shipping. Finally, we applied a qualitative RT-PCR method, which does not show the magnitude of expression of the given genes.

## Figures and Tables

**Table 1:** Sequence of oligonucleotide primers applied in this study

Gene name	Primer	Sequences 5’-3’
^*ESR*^ *1*	ESR1 forward	5´-GCCAAATTGTGTTTGATGGATTAA-3´
	ESR1 reverse	5´ GACAAAACCGAGTCACATCAGTAATAG-3´
*PGR*	PGR forward	5´-AGCTCATCAAGGCAATTGGTTT-3´
	PGR reverse	5´-ACAAGATCATGCAAGTTATCAAGAAGTT-3´
*ERBB2*	ERBB2 forward	5´-CCAGCCTTCGACAACCTCTATT-3´
	ERBB2 reverse	5´-TGCCGTAGGTGTCCCTTTG-3

**Table 2:** Socio-demographic characteristics of the study participants (n = 41)

Variables	Frequency
Sex	
Female	40 (97.6)
Male	1 (2.4)
Age group^a^	
< 30	1 (2.4)
30-39	20 (48.8)
40-49	11 (26.8)
50-59	5 (12.2)
≥ 60	3 (7.3)
Missing	1 (2.4)
Residential area	
Urban	22 (53.7)
Rural	12 (29.3)
Missing	7 (17.1)
Education level	
Illiterate	9 (22.0)
Primary school	14 (34.1)
High school	4 (9.8)
College and above	7 (17.1)
Missing	7 (17.1)

Values are presented as number (%). ^a^mean = 42.48 (standard deviation, 9.89) and median = 38.5 (range, 28-71). The sum of the percentages does not equal 100% because of rounding.

**Table 3:** Breast cancer risk factors of the study participants

Variables	Frequency
Menopausal status	
Premenopausal	24 (58.5)
Postmenopausal	13 (31.7)
Missing	4 (9.8)
Family history of breast cancer	
Yes	5 (12.2)
No	29 (70.7)
Missing	7 (17.1)
Radiation exposure to chest	
Yes	6 (14.6)
No	26 (63.4)
Missing	9 (22.0)
Physical exercise	
No	31 (75.6)
Occasionally	1 (2.4)
3 h/wk	1 (2.4)
5 h/wk	1 (2.4)
Other	1 (2.4)
Missing	6 (14.6)
Smoking	
Yes	0 (0.0)
No	35 (85.4)
Missing	6 (14.6)
Alcohol consumption	
Yes	1 (2.4)
No	34 (82.9)
Missing	6 (14.6)

Values are presented as number (%). The sum of the percentages does not equal 100% because of rounding.

**Table 4:** Clinico-pathologic characteristics of the study participants (n = 41)

Variables	Frequency
Stage at time of diagnosis	
0	1 (2.4)
I	0 (0.0)
II	14 (34.1)
III	21 (51.2)
IV	0 (0.0)
Missing	5 (12.2)
Histological type	
IDC	37 (90.2)
ILC	1 (2.4)
Mixed	1 (2.4)
DCIS	1 (2.4)
Micropapilary	1 (2.4)
Histological grade	
I	1 (2.4)
II	16 (39.0)
III	23 (56.1)
Missing	1 (2.4)
Sites of breast cancer	
Right	15 (36.6)
Left	24 (58.5)
Missing	2 (4.9)

Values are presented as number (%). IDC, invasive ductal carcinoma; ILC, invasive lobular carcinoma; DCIS, ductal calcinoma in situ. The sum of the percentages does not equal 100% because of rounding.

**Table 5:** Evaluation of ER, PR, HER2, and Ki-67 using immunohistochemistry (n = 41)

Variables	Frequency
ER	
Positive	38 (92.7)
Negative	3 (7.3)
PR	
Positive	27 (65.9)
Negative	14 (34.1)
HER2	
Positive	8 (19.5)
Negative	33 (80.5)
Ki-67	
High	25 (61.0)
Low	16 (39.0)

Values are presented as number (%). ER, estrogen receptor; PR, progesterone receptor; HER2, human epidermal growth factor receptor 2.

**Table 6:** Evaluation of ER, PR, and HER2 using endpoint RT-PCR (n = 41)

Variable	Endpoint RT-PCR	Frequency
ER	Positive	29 (70.7)
	Negative	12 (29.3)
PR	Positive	5 (12.2)
	Negative	36 (87.8)
HER2	Positive	9 (22.0)
	Negative	32 (78.0)

Values are presented as number (%). RT-PCR, reverse transcription PCR; ER, estrogen receptor; PR, progesterone receptor; HER2, human epidermal growth factor receptor 2.

**Table 7:** IHC and endpoint RT-PCR for molecular subtyping of breast cancer (n = 41)

Molecular subtypes	Based on IHC			Based on endpoint RT-PCR
	Using the expression of ER, PR and HER2	Using St. Galen 2013 classification (ER, PR, HER2 & Ki-67)		Using the expression of ER, PR and HER2
	Frequency	Frequency		Frequency
Luminal-A	31 (75.6)	14 (34.1)		21 (51.2)
Luminal-B	7 (17.1)	24 (58.5)		8 (19.5)
TNBC	2 (4.9)	2 (4.9)		11 (26.8)
HER2+	1 (2.4)	1 (2.4)		1 (2.4)

Values are presented as number (%). IHC, immunohistochemistry; RT-PCR, reverse transcription PCR; ER, estrogen receptor; PR, progesterone receptor; TNBC, triple negative breast cancer; HER2, human epidermal growth factor receptor 2; +, positive. The sum of the percentages does not equal 100% because of rounding.

**Table 8:** Concordance between IHC and endpoint RT-PCR based on ER, PR, and HER2 status

Variables	Concordance				
	OPA	PPA	NPA	κ value	*P*-value
ER	68.3 (28/41)	71.1 (27/38)	33.3 (1/3)	0.018 (< 0.20)	> 0.05
PR	39.0 (16/41)	14.3 (4/24)	92.3 (12/13)	0.045 (< 0.20)	> 0.05
HER2	82.9 (34/41)	62.5 (5/8)	87.9 (29/33)	0.481 (0.41-0.60)	< 0.05

Values are presented as % (number/number). IHC, immunohistochemistry; OPA, overall percent agreement; PPA, positive percent agreement; NPA, negative percent agreement; RT-PCR, reverse transcription PCR; ER, estrogen receptor; PR, progesterone receptor; HER2, human epidermal growth factor receptor 2.

**Table 9:** Concordance between IHC and endpoint RT-PCR based on molecular subtypes

IHC based	Endpoint RT-PCR based				κ value	OPA
	Luminal-A	Luminal-B	HER2+	TNBC		
Luminal-A	19 (61.3)	4 (12.9)	0 (0.0)	8 (25.8)	0.200*	56.1 (23/41)
Luminal-B	2 (28.6)	3 (42.9)	1 (14.3)	1 (14.3)		
HER2+	0 (0.0)	1 (14.3)	0 (0.0)	0 (0.0)		
TNBC	1 (50.0)	0 (0.0)	0 (0.0)	1 (50.0)		
Total	22 (53.7)	8 (19.5)	1 (2.4)	10 (24.4)		

Values are presented as number (%) or % (number/number). IHC, immunohistochemistry; OPA, overall percent agreement; RT-PCR, reverse transcription PCR; TNBC, triple negative breast cancer; HER2, human epidermal growth factor receptor 2; +, positive. The sum of the percentages does not equal 100% because of rounding. *P < 0.05.
